# Is there an association between full IQ score and mental health problems in young adults? A study with a convenience sample

**DOI:** 10.1186/s40359-020-0372-2

**Published:** 2020-01-30

**Authors:** Linde Melby, Marit S. Indredavik, Gro Løhaugen, Ann Mari Brubakk, Jon Skranes, Torstein Vik

**Affiliations:** 1 Faculty of Medicine and Health Sciences, Norwegian University of Science and Technology (NTNU), 6630 Tingvoll, Norway; 2 Child and Adolescent Psychiatry, Department of Clinical and Molecular Medicine, Faculty of Medicine and Health Sciences, NTNU, Trondheim, Norway; 3 Department of Pediatrics, Sørlandet Hospital Arendal, Arendal, Norway; 4 Pediatrics, Department of Clinical and Molecular medicine, Faculty of Medicine and Health Sciences, NTNU, Trondheim, Norway; 5 St. Olavs Hospital, Trondheim, Norway

**Keywords:** Intelligence quotient, Borderline intellectual functioning, Mental health, ADHD

## Abstract

**Background:**

Intelligence is the aggregate or global capacity of the individual to act purposefully, to think rationally and to deal effectively with the environment. Previous studies have shown that individuals with intellectual disability, IQ < 70, have increased risk of being diagnosed with one or more mental disorders. We wanted to investigate if this also applies to individuals with IQ between 70 and 85.

**Methods:**

In this study, data was abstracted from a longitudinal follow-up study of individuals with low birth weight and a control group. In the present study, mental health of participants with borderline IQ, defined as a full IQ score 70–84, were compared with mental health of a reference group with full IQ scores ≥85. Mental health at age 19 was assessed using the Schedule for Affective Disorder and Schizophrenia for School-age Children Present and Lifetime (K-SADS P/L) whereby scores meeting the diagnostic criteria for a mental disorder were defined as having mental health problems. In addition the participants completed the ADHD-rating scale and the Autism Spectrum Quotient form (AQ). Logistic regression analyses were used to calculate odds ratio (OR) with 95% confidence intervals (CI) for high scores on the K-SADS.

**Results:**

Thirty participants with borderline IQ and 146 controls were included. Sixteen (53%) of the participants with borderline IQ met the diagnostic criteria on the K-SADS for any diagnosis compared with 18 (12%) in the reference group (OR: 6.2; CI: 2.6–14.9). In particular the participants with borderline IQ had excess risk of ADHD and anxiety. These associations were slightly attenuated when adjusted for birth weight and parents’ socioeconomic status.

**Conclusions:**

53% of the participants with borderline IQ had increased risk for a research assessed psychiatric diagnosis compared to about one in ten in the reference group. The group with borderline IQ also had higher total scores and higher scores on some sub-scores included in the Autism Spectrum Quotient form. Our results points towards an increased vulnerability for mental illness in individuals with borderline low IQ.

**Trial registration:**

The main study is recorded by the Regional Committee for Health Research Ethics in Mid-Norway (as project number 4.2005.2605).

## Background

Intelligence is the aggregate or global capacity of the individual to act purposefully, to think rationally and to deal effectively with the environment [1]. Intelligence quotient (IQ) is a measure of general cognitive ability and is assessed by standardized tests, among them Wechsler Adult Intelligence Scale (WAIS) [2]. At the time of study, the WAIS 3rd edition was used [3]. An IQ score below 2 SD from mean value of 100, corresponding to a score below 70 is the main criteria for the diagnosis of mental retardation or intellectual disability, if accompanied by significant deficits in communication, self-care and social function necessary for independent daily functioning, according to the International Classification of Diseases (ICD-10) [4] and Diagnostic and Statistical Manual of mental disorders (DSM-IV) [5]. Full IQ scores of 70–84 indicate lower normal range IQ or borderline intellectual functioning [6].

In Norway, a white paper from the Norwegian parliament requires that the diagnosis of intellectual disability shall qualify for access to facilitated teaching, adapted work and special activities, and if needed, help in daily life activities [7]. In contrast, low average or borderline impaired IQ does not count as a separate diagnosis and consequently does not per se give access to the same rights. However, in a review of research during the last century, Gottfredson [8] reported that individuals with IQ below 85 tended to have low income jobs, requiring limited competence. Persons with IQ below 85 may therefore be at increased risk of financial problems and stress, which again may increase the vulnerability for mental health problems.

According to several studies, people with intellectual disability have increased risk of being diagnosed with a wide variety of psychiatric disorders [9–12]. In addition, Koenen et al. [9] found that IQ below 85 in childhood was associated with increased risk of schizophrenia later in life. The authors suggested that individual variability in the brain’s structure and function is responsible for cognitive reserve capacity, which may protect against psychopathology. Within the scope of the present study, full IQ score may be considered an important component of cognitive reserve capacity.

However, partly in contrast to this interpretation, implying that moderately low IQ could be a component in a causal chain leading to mental health problems, low IQ and psychiatric disorders may in fact have the same causes. For example, an early brain injury may lead to IQ < 85 as well as to mental health problems [13, 14]. This interpretation is proposed in some studies on mental health problems in very- and extremely preterm born individuals. Thus, an IQ below the normal range may both be in the causal chain, as well as have similar causes as mental health problems.

Regardless of these interpretations, mental health problems may not appear before the child starts school [15, 16]. At this age, some of these children may experience learning disorders, hyperactivity or attention deficit, and develop behaviour problems or even drop out of school [17].

The aim of this study was to investigate if borderline IQ (IQ score 70–84) was associated with a mental disorder at young adult age. We hypothesized that individuals with such IQ scores were more likely to have mental health problems than those with IQ above 85, and that this association was independent of low birth weight.

## Material and method

In this study with a convenience sample we used data abstracted from a longitudinal follow up study of children born preterm with birth weight below 1500 g, or at term as small for gestational age (birth weight below the 10th percentile adjusted for parity, sex and gestational age), and a randomly selected control group comprising children born at term with birth weight above the 10th percentile [18]. The study participants were born during 1986–1988, and they were IQ-tested at 5, 14 and 19 years, and their mental health was assessed at age 14 and 19 years.

In the present study we used information on full IQ score and mental health collected between 2005 and 2007 when the participants were 19 years old.

## Variables

### Exposure variable

The Intelligence Quotient (IQ) was assessed using Wechsler Adult Intelligence Scale III (WAIS-III) [2, 19] by an experienced neuropsychologist, blinded to the child’s mental health status and birth weight group. The test consists of 13 subtests, of which eleven is used in the calculation of IQ scores. In addition to Full, Verbal and Performance IQ, four indices were calculated: Verbal comprehension, Perceptual organization, Working memory and Processing speed. Mean IQ score is set at 100 in the normative sample. Scores between 85 and 115 (+/− 1 Standard Deviation; SD) are considered the average range according to the normal distribution curve. In this study we have defined IQ-score 70–84 as” borderline IQ”. Participants with borderline IQ were compared with a reference group where the participants had IQ scores of 85 or above.

### Outcome variable

In this longitudinal follow-up between 14 and 19 years, psychiatric diagnoses were assessed with the validated instrument Schedule for Affective Disorder and Schizophrenia for School-age Children Present and Lifetime version (K-SADS P/L) [20, 21]. The Norwegian translation of the K-SADS P/L has been validated in school-aged children [22] and in Norwegian children with Obsessive Compulsive Disorder as part of a Nordic study [23]. The instrument is widely used in research and clinical practice in Norway [24], and has been further validated in other Nordic countries, with similar languages and medical care [25, 26]. An experienced child psychiatrist, unaware of the child’s IQ scores and birth weight group, conducted this semi-structured interview [27]. The interview consists of two parts, where the first part comprises a number of screening questions, while in the second part these questions are supplemented with specific questions according to diagnostic criteria of Diagnostic and Statistical Manual of Mental Disorders-IV-TR (DSM-IV-TR) [5]. The interviews are usually made with both the child and its parents present, but at this 19-year follow-up assessment only the young adults themselves were interviewed. Although originally designed for children and adolescents up to 18 years, it has been applied to older individuals in other studies [28, 29].

The clinician summarised the information and classified the severity of symptoms as 1) those who met the criteria for a psychiatric diagnosis, 2) those who had subthreshold symptoms (≥ 75% of diagnostic criteria fulfilled), or 3) those without a psychiatric diagnosis or subthreshold symptoms. In this study, only those who met the criteria for a psychiatric diagnosis were defined as having mental health problems.

In addition to evaluating if the participants’ symptoms met the specific criteria for an anxiety disorder, attention deficit/hyperactivity disorder (ADHD) and/or mood disorder, we dichotomized the study population in those who met the criteria of “any” psychiatric disorder across the range of all disorders assessed in the K-SADS P/L interview, and those with no such diagnosis.

### Secondary outcomes: symptoms of autism and ADHD

Symptoms of autism were recorded using the Autism Spectrum Quotient (AQ) [30] completed by the participants themselves. This widely used screening tool is designed for individuals with Asperger Syndrome (AS) or highly functioning persons with autism. The questionnaire consists of 50 items with yes/no answers, and the scores are grouped in five areas: social skills, attention switching, attention to detail, communication and imagination. The maximum score is 50, and scores above 32 suggest autistic traits [31, 32]. The English version of the questionnaire has good validity [33]. The main author of the test approved a translation into Norwegian by consultants in child and adolescent psychiatry, but the Norwegian version has not been validated in this population (personal communication with the translator). The tool is recommended for use in persons with IQ above 85 due to the relatively advanced requirements for reading skills and understanding. As autistic spectrum traits are not screened in the K-SADS interview, we chose to include this instrument, although with reservations to the results.

Based upon the low prevalence of autism spectrum disorders, we expected that few participants would have scores above 32, and we therefore divided the participants into two groups based on the median value in the normal birth weight group. A” high score”, suggesting more symptoms, was defined as an AQ-score above the median, while a” low score” was defined as a score at or below the median.

To supplement the diagnostic evaluation of ADHD according to the K-SADS P/L interview, symptoms of ADHD were recorded using the questionnaire ADHD-Rating Scale IV (ADHD-RS) [34], screening for symptoms during the last 6 months, completed by the participants themselves. The questions correspond to the 18 criteria for ADHD in DSM-IV, and each question is rated from 0 to 3, based on the frequency of the asked events, and higher scores indicate more symptoms. The total score is divided into two sub-categories: attention deficit and hyperactivity/impulsiveness. The questionnaire has been tested in multiple studies, both in Europe and USA, and has been shown to have good reliability and validity [35].

Based on the median value of the normal birth weight group, we divided the participants into two groups. A” high score”, suggesting more symptoms, was defined as ADHD-RS-score above the median of the total study population, while a” low score” was defined as a score at or below the median.

### Other variables

Socioeconomic status (SES) was calculated using Hollingshead Two Factor Index of Social Position [36], based on both parents education and occupation, adjusted to today’s categories. This information was gathered at the 14-years assessment and supplemented at the 19-years follow-up for those who did not attend at 14 years. Information on SES was missing for 19 participants, 4 of 30 (13%) in the borderline IQ group and 14 of 146 (10%) in the reference group.

## Statistics

The IBM SPSS Statistics version 21 (SPSS Inc., Chicago, IL) was used for the statistical analysis. For the Autism Questionnaire and the ADHD-RS, inspecting histograms and Q-Q-plots assessed the distribution of the data. For variables with a normal distribution data are presented as mean and standard deviation (SD), while for variables with a non-normal distribution, data are presented as median and interquartile range.

Group differences in the distribution and median values of variables with non-normal distribution were compared using non-parametric test, including the Mann-Whitney U test. Percentages with 95% confidence intervals (CI) were calculated according to Newcombe and Altman [37]. Differences in proportions between groups were assessed using chi-square statistics or the Fisher’s exact test as appropriate. Binary logistic regression analyses were used to calculate crude odds ratios (OR) with CI, and OR with CI adjusted for possible confounders to assess if borderline IQ was associated with higher risk of psychiatric diagnoses and/or symptoms compared to participants in the reference group. In the multivariable analyses, variables that theoretically could affect the link between low IQ and mental health were considered as possible confounders. These variables included sex, SES, birth weight group, gestational age and mother’s age at birth. The multivariable analyses were completed with and without imputation of missing values for SES. In the latter case, the median value within each birth weight group was imputed. Possible confounders were included one at a time by following the hierarchic conceptual framework proposed by Victoria et al. [38]. Only confounders changing the unadjusted OR with more than 10% were included in the final models. Two-sided *p*-values below 0.05 were considered statistically significant.

### Ethics

In the main study the Regional Committee approved each follow-up examination for Medical and Health Research Ethics (REC) in Central Norway. For the examination at 19 years of age, providing the data in the present study, written informed consent was collected from all participants prior to participation in line with the approval for this specific follow-up examination (REC project number 4.2005.2605). At recruitment, all potential participants were considered competent to understand the information and to sign the informed consent form. For the examination at 15 years (socioeconomic status), written informed consent was obtained from the parents while the adolescents assented to participate. All participants were offered neuropsychological feedback to discuss the results of their cognitive assessment, and if an undiagnosed mental disorder was suspected, or in case of unmet health needs, the participants were offered referral to the appropriate health services.

## Results

Of the 178 participants who had their IQ as well as their mental health assessed at age 19 years, 146 (82%) had full IQ equal to or above 85, and 32 (18%) had full IQ below 85. Two participants had full IQ below 70 and were excluded from further analyses. Thus, the study population comprised 30 participants with borderline IQ, and 146 controls in the reference group. Twelve of the participants in the reference group had IQ above 115.

Some background information is shown in Table 1. Of the 146 participants in the reference group, 80 (55%) were women, while among the 30 participants with borderline IQ, 20 (67%) were women. As expected, the parents of the participants in the borderline intellectual functioning group had lower SES, and the participants themselves had needed more special education at school compared with the reference group (Table 1). The table also shows the proportion of participants recruited from each of the birth weight groups in the longitudinal follow-up study.

**Table 1:** Background characteristics of 30 participants with borderline IQ (i.e. full IQ score 70–84) and 146 participants in the reference group (i.e. full IQ equal to or above 85)

	Borderline IQ	Reference group
Parental socioeconomic status^a^	N (%)	N (%)
I -II	8 (31%)	26 (20%)
III	13 (50%)	27 (20%)
IV-V	5 (19%)	79 (60%)
Female sex	20 (67%)	80 (55%)
In need of special education	12 (40%)	5 (3%)
Birth weight group^b^		
Very low birth weight (i.e. < 1500 g	15 (50%)	34 (23%)
Term small for gestational age (< 10th centile)	9 (30%)	44 (30%)
Term born control group	6 (20%)	68 (47%)
Mean age (SD) in years at testing with K-SADS P/L	19.2 (0.5)	19.3 (0.8)

^a^SES missing in four (13%) individuals in the Borderline IQ group and in 14 (10%) individuals in the reference group

^b^According to the main study

Table 2 shows that 16 (53%) of the participants with borderline IQ met the criteria for a psychiatric diagnosis. In total, five (17%) had ADHD, seven (23%) had an anxiety disorder, one (3%) had a mood disorder and eight (27%) had “other”, not further specified diagnoses. These proportions were significantly higher compared to the reference group (Table 2). Three participants in the borderline IQ group suffered from both ADHD and anxiety, and two had ADHD and “other” psychiatric diagnoses. Two participants suffered from anxiety and “other” psychiatric diagnosis, and one had ADHD, anxiety and “other” psychiatric diagnosis. Thus, eight participants with borderline IQ had more than one diagnosis, while this was the case for only one participant in the reference group. The proportion with mood disorders did not differ between the two groups. Among the 12 participants with IQ of 115 or above, none had a psychiatric diagnosis.

**Table 2:** Psychiatric diagnoses assessed with the K-SADS P/L at age 19 in participants with borderline IQ (IQ scores between 70 and 84) and in a reference group (IQ scores equal to or above 85)

	Borderline IQ		Reference group		*p*-value
	n	% (CI)^a^	n	% (CI)^a^	
Any psychiatric disorder	16/30	53 (36–70)	18/146	12 (8–19)	< 0.001
ADHD	5/30	17 (7–34)	4/146	3 (1–7)	0.002
Anxiety disorder	7/30	23 (12–41)	8/146	5 (3–10)	0.001
Mood disorder	1/30	3 (1–17)	6/146	4 (2–9)	0.843
Other diagnoses than ADHD, anxiety and mood disorder	8/30	27 (14–44)	1/146	1 (0–4)	< 0.001
Total	30/30	100	146/146	100	

^a^CI = 95% Confidence Intervall

Table 3 shows that the odds for meeting the criteria of any psychiatric disorder, for ADHD, anxiety and for “other disorders” were high in participants with borderline IQ compared with the reference group. The table also shows that birth weight group and SES attenuated these associations in the multivariable analyses, although the strength of the associations persisted. However, in the analyses without imputation of missing values for SES, the CI for ADHD overlapped 1.0, and for anxiety, the lower 95% CI was 1.0 (Table 3). If missing values for SES were imputed, the OR for ADHD adjusted for birth weight and SES was 6.0 (CI: 1.3–28.2) and for anxiety the adjusted OR was 4.3 (CI: 1.3–14.4). The other potential confounders did not affect the association between IQ and psychiatric diagnoses (data not shown).

**Table 3:** Unadjusted odds ratio (OR) with 95% confidence intervals (CI) for the association between borderline IQ and psychiatric diagnosis, and OR adjusted for birth weight group, parents’ socioeconomic status (SES) and both. The reference group is used as reference

	Unadjusted	Adjusted for birth weight group	Adjusted for SES^a^	Adjusted for SES and birth weight group^a^
	OR (CI)	OR (CI)	OR (CI)	OR (CI)
Any psychiatric diagnosis	6.2(2.6–14.9)	5.2(2.1–12.8)	5.0(1.9–13.1)	4.4 (1.6–11.7)
ADHD	7.1 (1.8–28.3)	4.8(1.1–20.0)	7.4 (1.5–37.6)	4.3 (0.8–22.5)
Anxiety	5.3 (1.7–15.9)	4.2(1.3–13.3)	4.1(1.2–13.8)	3.6 (1.0–12.3)
Mood	0.8 (0.1–6.9)	0.7(0.1–6.4)	0.7 (0.1–6.3)	0.7 (0.1–6.6)
Other	52.7 (6.3–442)	61.8 (6.8–562)	38.0(4.4–336)	49.0 (5.0–478)

^a^Socioeconomic status was missing in four (13%) participants in the borderline intellectual functioning group, and in 14 (9.6%) in the reference group. The adjusted odds ratios shown in the table are obtained in multivariable analyses not including participants with missing information on SES

Three participants in the reference group did not complete the AQ questionnaire. The group with borderline IQ had higher scores on social skills, communication, imagination and on the total AQ score, compared to the reference group whereas there were no differences in attention switching and attention to details between the two groups (Table 4).

**Table 4:** Scores (median and interquartile range (IQR)) on the Autism Questionnaire (AQ) self-report among 19-year old participants (*N* = 30) with borderline IQ (IQ scores between 70 and 84) and among 143 participants in a reference group with IQ scores equal to or above 85

	Borderline IQ			Reference group				
	Median	(IQR)	Mean rank	Median	(IQR)	Mean rank	Z	P*
Social skills	2	(1.0–3.0)	119.84	1	(0.0–2.0)	79.37	−4.12	< 0.001
Attention switching	4.5	(2.3–6.0)	87.64	4	(3.0–5.0)	85.68	−0.19	0.846
Details	3	(2.0–5.0)	82.80	4	(3.0–5.0)	86.63	−0.38	0.705
Communication	3	(1.3–4.0)	107.70	2	(1.0–3.0)	81.75	−2.58	0.010
Imagination	4	(3.0–5.0)	111.36	3	(2.0–4.0)	81.03	−3.01	0.003
Total	17	(13.0–20.8)	110.45	13	(10.0–17.0)	81.25	−2.86	0.004

*Mann-Whitney test for the differences in distribution between the groups

ADHD-RS completed by the participants themselves showed no statistically significant differences between the two groups, although there was a tendency towards higher scores on attention deficit and total score in the group with borderline IQ (Table 5).

**Table 5:** Median and interquartile range for scoring in ADHD-RS including total score in 19-year olds with borderline IQ (IQ scores between 70 and 84) and in a reference group (IQ scores equal to or above 85)

	Borderline IQ, n = 30		Reference group, *n* = 147		
	Median	IQR	Median	IQR	p
Attention deficit	5	(1.0–10.0)	3,5	(2.0–6.0)	0.298
Hyperactivity	4	(3.0–8.0)	4	(2.0–6.0)	0.211
Total	10	(4.5–17.0)	8	(5.0–11.0)	0.154

## Discussion

In this study we found that half of the participants with borderline low IQ (i.e. IQ between 70 and 85) at 19 years of age met the criteria for a psychiatric diagnosis, compared with only one in ten in the reference group. This implied an almost five times higher odds for a psychiatric disorder for participants with borderline IQ, compared to the reference group. ADHD and anxiety were the most common diagnoses among those with borderline IQ. Thus, our results suggest that young adults with borderline IQ have increased risk of mental health problems. The higher scores on the Autism Questionnaire in this group should be interpreted with caution since the absolute differences were small and since the questionnaire is recommended for people with IQ above 85.

The results showed a higher occurrence of ADHD in the borderline IQ group according to the K-SADS P/L interview, while when the participants rated their own symptoms by completing the ADHD-RS, there were no significant differences between the groups. The different results may be explained by the fact that the K-SADS interview conducted by an experienced child- and adolescent psychiatrist, allows for elaborative questions, which may increase the validity of the findings, while the ADHD-RS is a subjective assessment of various symptoms completed on a fixed questionnaire. Such differences between self-perceived symptoms and symptoms assessed by others have been described in several papers [13, 39].

It is unlikely that our main findings are due to chance, as indicated by the very low *p*-values for most of the differences between the groups. The groups did not differ significantly in scores on the self-reported ADHD-RS, however, it should be noted that the differences in attention deficit and total score were close to statistically significance (0.05–0.1).

A limitation of the present study is that it was completed in a convenience sample where participants born prematurely or with low birth weight at term were overrepresented. However, when we adjusted for birth weight group, the estimated odds ratio for mental health disorder among individuals with borderline IQ was still considerably increased. Other potential confounders, such as mother’s age and SES did not affect the association between low IQ and mental health disorders. Regarding the results obtained through the AQ questionnaire, a significant limitation is that the Norwegian version of this instrument has not been properly validated in a Norwegian population, and moreover, that the English version is validated for individuals with IQ above 85. Thus, although highly statistically significant, the small differences in median values between the low IQ and the reference group could have been caused by interpretation differences between the two groups.

An important strength of this study is that an experienced neuropsychologist and an experienced child and adolescent psychiatrist performed the assessments. They were blinded to earlier test results and also to each other’s test assessments, and to birth weight group. Also, the comprehensive assessment of mental health problems using the K-SADS P/L semi-structured interview, and the restrictive definition of mental health problems may be considered to strengthen the study. IQ score was calculated using a full WAIS. Thus it is unlikely that our findings are due to misclassification of cognitive functioning or mental health problems.

Our findings are consistent with several other studies [9, 40]. However, most previous studies have examined the risk for mental disorders among individuals with intellectual disability (IQ < 70) [10, 41, 42]. The Isle of Wight study found three to four times higher occurrence of psychiatric disease among participants with low IQ, defined as an IQ score below 70 compared to participants with normal IQ [40]. Similar results were reported by Borthwick-Duffy et al. [42], Strømme and Diseth [41] and Emerson [10]. The latter author also found a seven times higher risk for psychiatric disease among children with assumed intellectual disability. Emerson did not measure intellectual disability in the standard way, but defined intellectual disability as having learning disabilities, going to special schools or having mental age < 80% of chronological age. Emerson also found increased risk for any emotional disorders (OR 2.4, CI: 1.6–3.7), with post-traumatic stress disorder (PTSD) (OR 4.8, CI 1.1–20.9) being one of the largest contributors. Other findings included increased risk of generalized anxiety disorder (OR 2.6, CI 1.0–7.4) and depression (OR 1.7, CI 0.6–4.7) in the low IQ group [10].

Lamanna et al. found intellectual disability to be a risk factor for ADHD. They found that 22% of the participants with ADHD had coexisting intellectual disability [43]. Fernell E. et al. found that among adolescents diagnosed with ASD, 38% had intellectual disability [44].

The main findings of the present study may be considered consistent with all these studies. However, the rather novel finding in our study is that participants with borderline IQ seem to have as high a risk for psychiatric diagnoses as earlier studies have found among participants with intellectual disability. Our results may also be in keeping with one of the few studies in persons with low IQ defined as IQ below 85 [9]. In that study Koenen et al. found that high IQ protected against anxiety, social phobia and PTSD, while low IQ was a risk factor for anxiety, schizophrenia and depression [9].

There are at least two principally different plausible interpretations of the association between borderline IQ and the presence of a psychiatric disorder. First, IQ may be seen as an innate, largely genetically determined, and more or less constant trait [12], which however, may be modified by early environmental influences and/or global insults to the brain. A predisposition for different psychiatric disorders may also be inherited, i.e. be genetically determined, but the risk for psychiatric disorders may further be modified by early mal-development of the brain, such as seen in children born very preterm. Thus, it is possible that the association between below average IQ and psychiatric diagnosis do have some common cause. This connection is not unlikely, as neuro-developmental symptoms and disorders were present in our sample. This may partly explain the association in our population, since a large proportion of the individuals were born very preterm, and since others and we have shown that very preterm born individuals have high risk for cognitive and mental disorders, that may last into adulthood [13, 14, 27, 45]. Moreover, psychiatric disorders may be precipitated during childhood and adolescence by bullying, low self-esteem due to specific learning difficulties and physical deficits, or by other factors such as major life events; divorce, loss of a parent etc. [46].

An additional interpretation may therefore be that individuals with borderline IQ are more vulnerable to mental stress than individuals with average IQ. Thus, they may be more likely to develop a mental disorder as a consequence of their low IQ. This interpretation may be supported by the fact that the association between borderline IQ and mental health problems persisted when we adjusted for birth weight group, although residual confounding cannot be excluded. Moreover, this second interpretation would be in line with the notion that individuals with low IQ have reduced cognitive reserve capacity. Such reduced capacity may result in poor coping strategies, which can lead to mental health problems such as anxiety and depression [9]. Cognitive impairments will affect the individuals’ functioning in daily life and cause learning difficulties, while deficits in executive functions and attention may result in diagnoses such as ADHD/ADD. Poor academic and social coping strategies may lead to mental health disorders such as anxiety and depression [15, 17]. Problems with expressing their needs may lead to behaviour problems [9, 17]. This may become more visible as the individual gets older, especially when they start school and have to face increased demands from the environment.

The apparent dose-response relationship, whereby none of the participants with IQ above 115 had a psychiatric diagnosis, and the high ORs for diagnoses in the borderline IQ group compared to the reference group would be in line with the criteria proposed by Doll and Hill to indicate causal relationship [47]. Moreover, this interpretation may also be consistent with a timing aspect, since it may be argued that IQ is mainly determined in early childhood, while emotional disorders develop later in life [48].

Most likely the basis for the association between borderline IQ and psychiatric diagnosis is a combination of an early genetic and/or environmental influence on brain development leading both to low IQ as well as to increased risk for psychiatric disorders, and the higher vulnerability for mental stress among persons with limited cognitive reserves.

### Clinical implications

Regardless of whether low IQ is in the causal chain or if low IQ and mental disorders have a common aetiology, our findings have some important clinical implications. One implication is that kindergartens, schools and the society should pay particular attention to children and youths diagnosed with borderline IQ, being aware that these individuals are at increased risk of developing or having a psychiatric disorder. This may especially involve ADHD and anxiety disorders, and the risk of having more than one disorder. It is of particular importance to be aware that these children, often having learning difficulties, are also more likely to be bullied and develop low self-esteem [46]. However, even without early signs of delayed development or specific learning difficulties, individuals with borderline IQ may experience problems keeping up with the demands at school and in social interaction, as they grow older. Thus, schools should provide special tutoring and extra care to potentially prevent development of psychiatric disease also in children with borderline IQ, but without intellectual disability.

## Conclusion

In this study using research tools, we found that borderline IQ (IQ score 70–84) was associated with an increased risk of meeting the criteria for a psychiatric disorder, in young adulthood. The results have important clinical implications during childhood and adolescence that need attention.

## Data Availability

This data is collected from a still on-going longitudinal cohort study and therefore the data material is not yet freely available.

## Abbreviations

ADHD: Attention Deficit/Hyperactivity Disorder
AQ: Autism Quotient
IQ: Intellectual Quotient
K-SADS P/L: Schedule for Affective Disorder and Schizophrenia for School-age Children Present and Lifetime
SES: Socio Economic State
SGA: Small for Gestational Age
VLBW: Very Low Birth Weight
WAIS: Wechsler Adult Intelligence Scale
